# Assessing the accuracy and readability of ChatGPT 4.0's original and simplified responses to common patient questions regarding periacetabular osteotomy

**DOI:** 10.1002/jeo2.70457

**Published:** 2025-10-09

**Authors:** John M. Gaddis, Elias Arellano, Blake C. Martin, Yossef Alsabawi, Martin Salgado‐Flores, Charles South, Pablo Castaneda, Joel E. Wells

**Affiliations:** ^1^ University of Texas Rio Grande Valley School of Medicine Edinburg Texas USA; ^2^ Southern Methodist University Dallas Texas; ^3^ Texas Children's Hospital The Woodlands Texas USA; ^4^ Department of Orthopedic Surgery Baylor Scott and White Hip Preservation Center McKinney Texas USA

**Keywords:** artificial intelligence, ChatGPT, periacetabular osteotomy

## Abstract

**Purpose:**

The study aimed to evaluate the accuracy, comprehensiveness, and readability of responses generated by ChatGPT 4.0 to 30 common patient questions about the Bernese periacetabular osteotomy (PAO).

**Methods:**

Two fellowship‐trained orthopaedic surgeons specializing in hip preservation selected thirty questions from a prior study identifying common PAO questions on social media. Each question was entered into ChatGPT 4.0, and the surgeons independently graded responses. Responses were evaluated using an established grading system. Accuracy and comprehensiveness were assessed based on the concordance of response content with current literature. Readability was analysed by calculating the Flesch‐Kincaid Grade Level and Flesch‐Kincaid Reading Ease.

**Results:**

Regarding accuracy and comprehensiveness, 98.3% of responses were graded as “excellent” or “satisfactory, requiring minimal clarification.” Readability analysis revealed an average Flesch‐Kincaid Grade Level corresponding to an 11th‐grade reading level (11.09 ± 1.47) and a mean Reading Ease score requiring college level reading comprehension (39.12 ± 8.25) for original responses, 8th‐grade reading level (8.16 ± 1.46) requiring high school to college level reading comprehension (51.53 ± 9.62) for simplified responses, and 7th‐grade reading level (7.09 ± 1.23) requiring high school level reading comprehension (62.46 ± 7.48) for 6th grade responses.

**Conclusion:**

ChatGPT 4.0 offered excellent or satisfactory answers to the most common questions surrounding PAO. Asking ChatGPT 4.0 to simplify or respond at a specific reading level may increase the readability of responses. The 4.0 model has shown the potential to be a valuable adjunct for patient education, though the readability may need to be improved via simplified responses.

**Level of Evidence:**

Level N/A.

**Abbreviations**
AI: artificial intelligence
CI: confidence interval
PAO: periacetabular osteotomy
SD: standard deviation

## INTRODUCTION

As the use of the internet for health information grows, more patients are seeking online answers to their health‐related questions [37]. A product of this growth has been the development of ChatGPT (OpenAI), a generative artificial intelligence (AI) chatbot designed to provide written responses based on specific user prompts [8]. AI, specifically ChatGPT, has already begun to impact the medical field in clinical practice and research [8, 21], including specialties like orthopaedic surgery [9, 31, 32, 42].

The Bernese periacetabular osteotomy (PAO) is a hip‐preserving surgical technique for treating symptomatic hip dysplasia that decreases the chance of progression to end‐stage osteoarthritis while preserving function and enhancing hip joint longevity [7, 12, 14]. Frequently performed in young adults, the PAO has shown good short‐, intermediate‐, and long‐term outcomes [2, 7, 20, 26, 38]. Muir et al. showed that understanding the PAO is essential to patients; however, most patients had to rely on online resources because their surgeons only partially addressed their informational needs [25]. Furthermore, a study from Japan found information on the internet regarding PAOs needing to be of better quality and unreliable [36].

Previous studies have shown ChatGPT Version 3.5 provided satisfactory responses to questions regarding elbow ulnar collateral ligament reconstruction, total hip arthroplasty, and hip fractures [15, 24, 39]. Moreover, studies using the updated ChatGPT Version 4.0, which boasts enhanced reasoning, speed, and conciseness (https://openai.com/), proved this version of the updated AI chatbot could provide at least satisfactory responses to questions about hip arthroscopy, hip dysplasia, and PAO [19, 28]. To our knowledge, there is one study stating that ChatGPT 4.0 could provide satisfactory responses to questions about PAO, although the study did not analyse simplified or grade specific responses [19]. Therefore, this study aimed to determine the accuracy and readability of original, simplified, and grade specific responses provided by ChatGPT 4.0 of 30 common questions asked by patients concerning PAO. The use of AI and ChatGPT could potentially be a means of addressing informational gaps by providing patients with accurate and detailed information regarding PAOs. Based on results from previous studies [15, 24, 28, 39], we hypothesised that ChatGPT 4.0's original, simplified, and grade specific responses would provide satisfactory answers to most, if not all, common questions about PAO requiring minimal‐to‐no clarification. However, we believe the responses will be written at excessive reading levels, making them more difficult to full comprehend, even when prompted to simplify or write the responses at a specific reading level.

## METHODS

One study previously described the most commonly asked questions by the PAO population on social media [11]. Based on the results from this study [11], a list of 48 widely asked pre‐ and postoperative questions was developed. Two fellowship‐trained orthopaedic surgeons specializing in hip preservation (JW and PC) reviewed and selected 30 questions for analysis they frequently encounter in clinical practice and deemed most applicable. On 9 October 2024, each question was individually entered into ChatGPT Version 4.0 by author JG without follow‐up questions or duplicate queries (https://openai.com/). Original responses were individually pasted back into ChatGPT by author JG using the prompts “Please simplify this response so it is easier to understand” or “Please write this response at a sixth grade reading level” for a total of 90 responses: 30 original, 30 simplified, and 30 sixth grade. Responses were saved in Microsoft Excel and sent to the surgeons (JW and PC) for independent grading. Prompts were blinded from the reviewers (JW and PC) to limit potential bias in grading.

Following the methodology of similar studies [15, 24, 28, 39], each answer was graded using the rating system established by Mika et al. [24] (Table 1), which has been previously used to assess responses from ChatGPT to questions relating to elbow ulnar collateral ligament reconstruction [15], total hip arthroplasty [24], hip fracture [39], and hip arthroscopy [28]. Both author surgeons (JW and PC) individually assessed and graded each answer using an evidence‐based approach and the rating system developed by Mika et al., with ratings being either “excellent response not requiring clarification,” “Satisfactory requiring minimal clarification,” “Satisfactory requiring moderate clarification,” or “Unsatisfactory requiring substantial clarification” [24]. Excellent responses were considered accurate, comprehensive, and in line with current literature and guidelines. Satisfactory responses needing minimal clarification were still deemed to be accurate, but they lacked additional information needed to completely answer the question prompted. Satisfactory responses requiring moderate clarification were still considered accurate but contained outdated or irrelevant information about the question. Unsatisfactory responses were answers containing inaccurate or overly vague information that could potentially lead to misinterpretation.

**Table 1:** Grading system used to evaluate ChatGPT responses.

Score	Response accuracy description
1	Excellent response not requiring clarification
2	Satisfactory requiring minimal clarification
3	Satisfactory requiring moderate clarification
4	Unsatisfactory requiring substantial clarification

The responses from ChatGPT were passed directly into the free online Good Calculators' Flesch‐Kincaid Calculator to determine the readability of each response [10]. The Flesch‐Kincaid formulas have been previously used by multiple studies to assess the reading ease and grade level of written material which provides a measure of how difficult it is to understand [3, 4, 10, 15, 27, 33]. The purpose of analyzing the readability of responses was to determine if they were written at a level that could be best understood by patients, which the National Institutes of Health and the American Medical Association recommends being at a sixth grade reading level or lower [3, 13, 29, 33]. The Flesch‐Kincaid Grade Level indicates the educational level required to understand a specific body of text [10]. A higher score correlates with a text that is more difficult to comprehend (Ex. A Flesch‐Kincaid Grade Level of eight requires the reader to have reached the eighth grade to understand the text without difficulty). A description of the Flesch‐Kincaide formulas are shown in Table 2.

**Table 2:** Formulas for Flesch Kincaid reading ease and grade level.

Flesch Kincaid Reading Ease:	= 206.835 – 1.015 × (Total Words/Total Sentences) – 84.6 × (Total Syllables/Total Words)
Flesch Kincaid Grade Level:	= 0.39 × (Total Words/Total Sentences) + 11.8 × (Total Syllables/Total Words) – 15.59

The Flesch‐Kincaid Reading Ease shows how easy reading the written material is and is scored from zero (highly difficult to read) to 100 (extremely easy to read) [10]. Furthermore, the Flesch‐Kincaid Reading Ease can also predict the grade level required to understand a particular passage, such that a Reading Ease score of 60–70 correlates with eighth‐ and ninth‐grade reading levels, 50–60 with high school reading levels, 30–50 with college reading levels, and below 30 with college graduate reading levels [10]. The formulas for the Flesch‐Kincaid Grade Level and Reading Ease are shown in Table 2. The number of sentences and words of each response, along with the average words per sentence and average syllables per word, were recorded to analyse the structure and complexity of responses further.

All data analysis was conducted using Anaconda Navigator 2.6.4 and Jupyter Notebook 6.4.5 with Python 3.9., using the pandas, SciPy, and scikit‐learn libraries. Quantitative variables were summarised using the mean and standard deviation (SD). Inter‐rater reliability for categorical scores was assessed using Cohen's Kappa. Statistical significance with a 95% confidence interval (CI) was evaluated and set at *α* = 0.05.

## RESULTS

Thirty questions related to PAO were included in the study and individually entered into ChatGPT 4.0. The complete list of questions and responses can be found in Supporting Information: Table 1. Each response contained an introductory paragraph, while 22 (73%) included a summary or conclusion paragraph.

### Response accuracy

**Figure 1:**
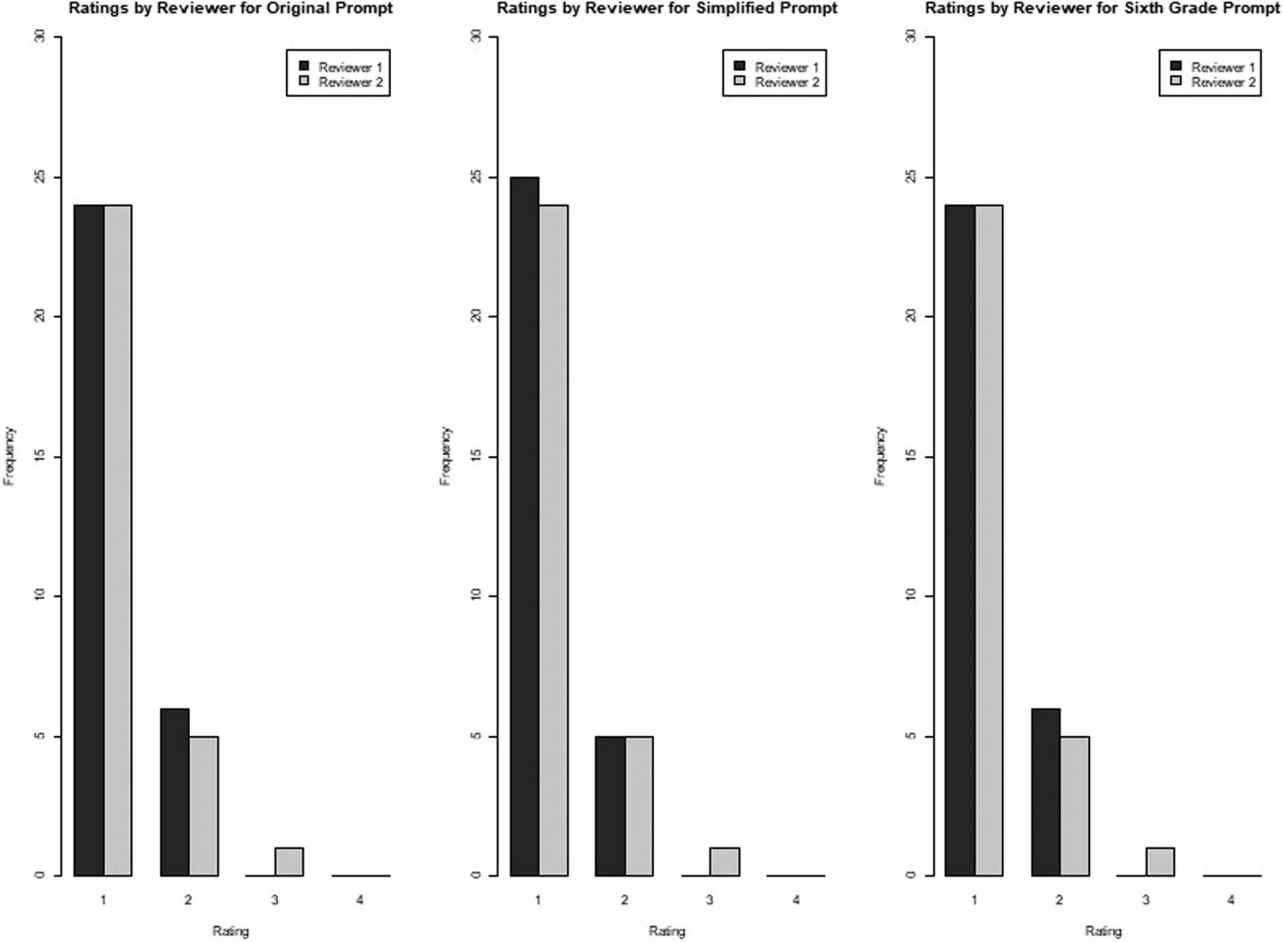
Ratings by reviewer for the three different question prompts.

**Figure 2:**
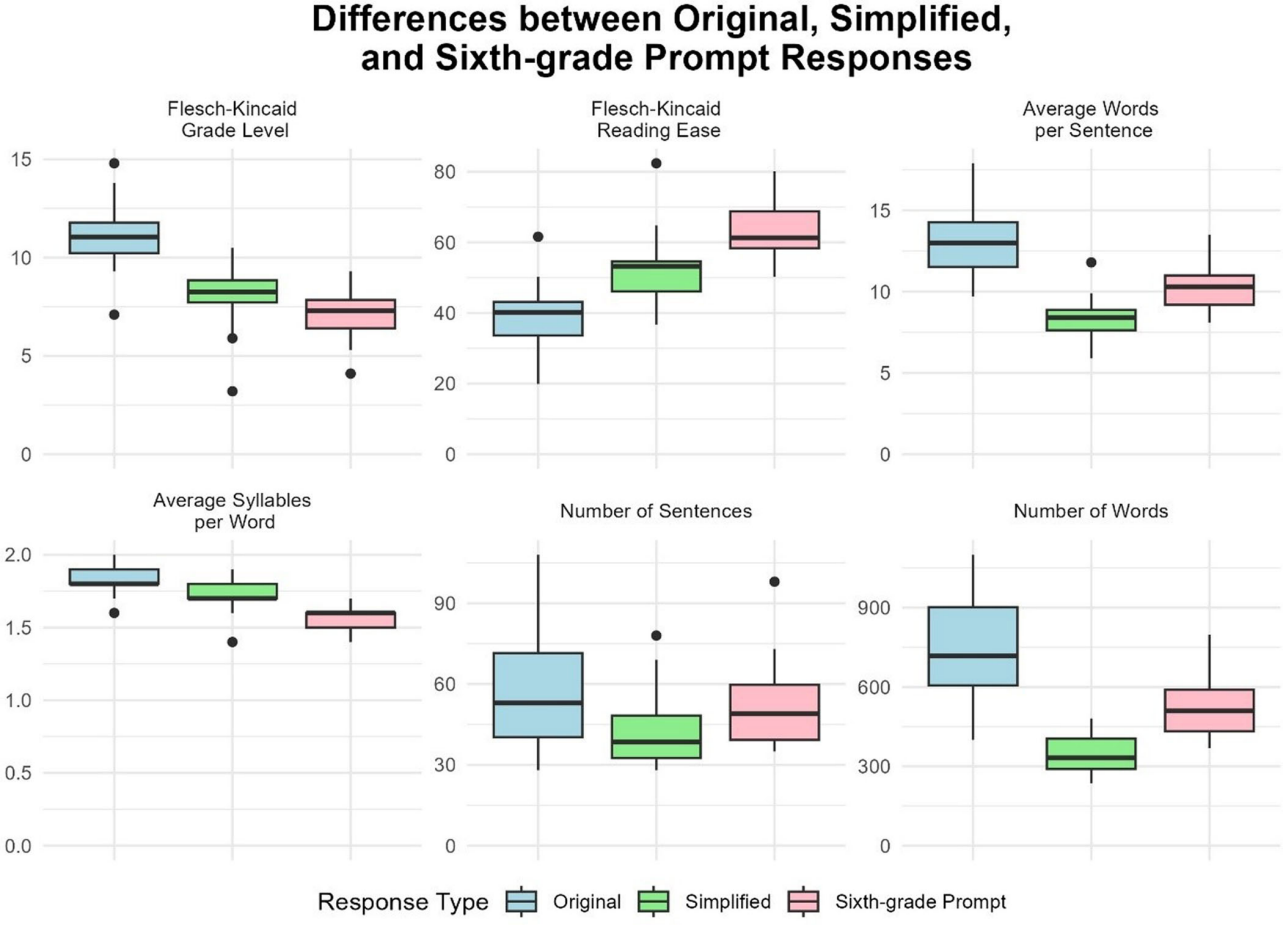
Differences between the three different prompt responses.

For original responses, Reviewer 1 and Reviewer 2 scored 24 (80%) and 17 (56.7%) responses, respectively, as “1, Excellent response not requiring clarification.” The remaining responses were graded as ‘satisfactory’ with six (20%) and 12 (40%) only requiring minimal clarification by Reviewer 1 and Reviewer 2, respectively. One (3.3%) response required moderate clarification by Reviewer 2 (“What is the likelihood of a fracture after periacetabular osteotomy?”); no responses were graded as “4, Unsatisfactory requiring substantial clarification.” The reviewer score average for all responses was 1.2 ± 0.41 and 1.47 ± 0.57 for reviewers 1 and 2, respectively (Tables 3 and 4 and Figures 1 and 2).

**Table 3:** Contingency tables.

Original		Reviewer 2 scores									
		1	2	3	4						
Reviewer 1 scores	1	17	6	1	0
	2	0	6	0	0
	3	0	0	0	0
	4	0	0	0	0

Simplified		Reviewer 2 scores				Sixth grade		Reviewer 2 scores			
		1	2	3	4			1	2	3	4
Reviewer 1 scores	1	20	4	1	0	Reviewer 1 scores	1	21	2	1	0
	2	4	1	0	0		2	3	3	0	0
	3	0	0	0	0		3	0	0	0	0
	4	0	0	0	0		4	0	0	0	0

*Note*: Contingency tables showing the level of agreement between the two reviewers by each prompt type.

**Table 4:** Mean scores, readability and sentence structure of responses from ChatGPT 4.0 by prompt type.

	Original		Simplified		Sixth grade	
Readability metrics	Mean ± SD	(Min, Max)	Mean ± SD	(Min, Max)	Mean ± SD	(Min, Max)
Reviewer 1	1.2 ± 0.41	(1, 2)	1.17 ± 0.38	(1, 2)	1.2 ± 0.41	(1, 2)
Reviewer 2	1.47 ± 0.57	(1, 3)	1.23 ± 0.5	(1, 3)	1.23 ± 0.5	(1, 3)
Flesch Kincaid Grade Level	11.09 ± 1.47	(7.1, 14.8)	8.16 ± 1.46	(3.2, 10.5)	7.09 ± 1.23	(4.1, 9.3)
Flesch Kincaid Reading Ease	39.12 ± 8.25	(19.9, 61.6)	51.53 ± 9.62	(36.7, 82.4)	62.46 ± 7.48	(50.3, 80.2)
Average words per sentence	13.25 ± 2.22	(9.7, 17.9)	8.36 ± 1.16	(5.9, 11.8)	10.27 ± 1.41	(8.1, 13.5)
Average syllables per word	1.82 ± 0.09	(1.6, 2)	1.74 ± 0.11	(1.4, 1.9)	1.58 ± 0.08	(1.4, 1.7)
Number of sentences	58.00 ± 22.39	(28, 108)	42.20 ± 12.79	(28, 78)	51.67 ± 14.61	(35, 98)
Number of words	739.07 ± 219.96	(400, 1100)	342.67 ± 68.35	(236, 481)	518.20 ± 105.37	(368, 798)

Abbreviation: SD, standard deviation.

For simplified responses, Reviewer 1 and Reviewer 2 scored 25 (83.3%) and 24 (80%) responses, respectively, as “1, Excellent response not requiring clarification.” The remaining responses were graded as ‘satisfactory’ with five (16.7%) and five (16.7%) only requiring minimal clarification by Reviewer 1 and Reviewer 2, respectively. One (3.3%) response required moderate clarification by Reviewer 2 (“What is the likelihood of a fracture after periacetabular osteotomy?”); no responses were graded as “4, Unsatisfactory requiring substantial clarification.” The reviewer score average for all responses was 1.17 ± 0.38 and 1.23 ± 0.5 for reviewers 1 and 2, respectively (Tables 3 and 4 and Figures 1 and 2).

For sixth grade responses, Reviewer 1 and Reviewer 2 scored 24 (80%) and 24 (80%) responses, respectively, as “1, Excellent response not requiring clarification.” The remaining responses were graded as ‘satisfactory’ with six (20%) and five (16.7%) only requiring minimal clarification by Reviewer 1 and Reviewer 2, respectively. One (3.3%) response required moderate clarification by Reviewer 2 (“What is the likelihood of a fracture after periacetabular osteotomy?”); no responses were graded as “4, Unsatisfactory requiring substantial clarification.” The reviewer score average for all responses was 1.2 ± 0.41 and 1.23 ± 0.5 for reviewers 1 and 2, respectively (Tables 3 and 4 and Figures 1 and 2).

Cohen kappa was used to determine interrater reliability and was found to be *κ* = 0.5 (*p* = 0.003, 95% CI [0.206–0.791]), indicating “moderate” agreement between the reviewers [23]. The disagreement between reviewers was mainly seen when determining if a response was “1, Excellent response not requiring clarification” or “2, Satisfactory requiring minimal clarification” (*n* = 6, 20%). The question “What is the likelihood of a fracture after periacetabular osteotomy?” was the largest source of disagreement between the surgeons – rated “1, Excellent response not requiring clarification” by Reviewer 1 and “3, Satisfactory requiring moderate clarification” by Reviewer 2.

### Response readability

For original responses, the average Flesch‐Kincaid Grade Level for all responses was 11.09 ± 1.47 which correlates approximately to the reading level of a high school junior. The Flesch‐Kincaid Reading Ease for all responses averaged 39.12 ± 8.25 (college level). Twenty‐eight responses (93.3%) required at least a college‐level education to comprehend (Flesch‐Kincaid Grade Level ≥ 12.5 or Flesch‐Kincaid Reading Ease ≤ 50.0). Responses from ChatGPT 4.0 contained an average of 58.00 ± 22.39 sentences and 739.07 ± 219.96 words. These results can be seen in Table 4.

For simplified responses, the average Flesch‐Kincaid Grade Level for all responses was 8.16 ± 1.46 which correlates approximately to the reading level of an eighth grader. The Flesch‐Kincaid Reading Ease for all responses averaged 51.53 ± 9.62 (high school to college level). Responses from ChatGPT 4.0 contained an average of 42.20 ± 12.79 sentences and 342.67 ± 68.35 words. These results can be seen in Table 4.

For sixth grade responses, the average Flesch‐Kincaid Grade Level for all responses was 7.09 ± 1.23 which correlates approximately to the reading level of a seventh grader. The Flesch‐Kincaid Reading Ease for all responses averaged 62.46 ± 7.48 (high school level). Responses from ChatGPT 4.0 contained an average of 51.67 ± 14.61 sentences and 518.20 ± 105.37 words. These results can be seen in Table 4.

## DISCUSSION

ChatGPT, an AI chatbot, continues to grow significantly in popularity and use. More specifically, its use is being studied in orthopaedic surgery for various tasks, including answering common questions related to specific surgeries [15, 24, 28, 32, 39], aiding with diagnosis [18], and assisting with clinical documentation [5, 21]. Therefore, we sought to analyse ChatGPT 4.0's responses to common questions regarding PAO to determine if this AI software could provide accurate and relevant responses to patients. Additionally, we aimed to examine ChatGPT 4.0's ability to simplify its responses to improve the reading level while maintaining accuracy.

ChatGPT was able to give excellent or satisfactory responses to all questions given regarding PAO with most requiring minimal or no clarification (98.3%), including responses that were simplified using specific prompts. These findings disproved our initial hypothesis as most responses were graded as ‘excellent’ rather than ‘satisfactory.’ However, none of the responses were noted as ‘unsatisfactory,’ thus indicating the potential for this software to be used as an educational resource concerning PAO. Future studies assessing ChatGPT 4.0's responses to more complex patient questions are needed to better understand the limitations of the AI programme in providing sophisticated medical information.

Similar to other studies [15, 32, 40], the original responses generated by ChatGPT in our research were written at much higher educational levels than the sixth grade reading level recommended by the National Institutes of Health and the American Medical Association [13, 29]. Excessively high reading levels needed to comprehend these responses could potentially limit their applicability to a PAO population. PAO is commonly performed in younger individuals with hip dysplasia; [12, 14] therefore, it is essential to determine if online resources, such as ChatGPT, present easily understandable information for patients.

A study analysing ChatGPT's responses to common questions about total hip arthroplasty and total knee arthroplasty found that the authors were able to significantly decrease the Flesch‐Kincaid Grade Level from 12.15 to 11.14 by telling the software “Please explain so it is easier to understand” [40]. Although the Flesch‐Kincaid Grade Level of the previous study's simplified answers was still higher than the average for our three different responses, asking ChatGPT to simplify responses to questions regarding PAO significantly improved the readability of our responses, albeit to a much larger degree than the previous study described [40]. However, another study showed that both detailed and simplified responses regarding questions on osteoarthritis easily exceeded a 6^th^ grade reading level and there was no significant difference in readability between the groups receiving detailed versus simplified instructions [41]. One notable difference between our study and this previous study is the previous study used ChatGPT 3.5 compared to our study which used ChatGPT 4.0 [41]. This could help explain the lack of improvement in readability in that study when compared to ours. However, another study on ChatGPT responses to questions about fragility fractures showed an improvement in readability when requesting the questions be answered in “plain language” compared to without this prompt [1]. This study also used ChatGPT 3.5 [1]. The findings from this study and previous studies studying various forms of simplification show the potential variation of ChatGPT's responses for both versions of the AI chatbot (3.5 or 4.0) [1, 40, 41]. These differences could potentially be due to the way in which ChatGPT is asked to simplify responses such as “simplify” or “use plain language” [1, 41]. Another explanation for readability variation is the topic of question. Topics that are less complex might allow ChatGPT to respond with answers that are easier to read and comprehend when compared to subjects that are more complex. A difference in the amount of information available or the comprehension variability of various topics are possible explanations for this difference in readability. Even still, it remains to be determined if these simplified responses using ChatGPT 3.5 would maintain the accuracy and completeness needed to be considered ‘excellent’ or ‘satisfactory’ such as the simplified responses in our study using ChatGPT 4.0.

While many previous studies assess ChatGPT's responses to common orthopaedic conditions or procedures, most have utilised the older, free‐to‐use ChatGPT 3.5 [15, 18, 24, 35, 39]. In this study, we used ChatGPT 4.0, the newest and most advanced version, which requires a paid subscription to access. Multiple studies that compared the capabilities of ChatGPT 4.0 to ChatGPT 3.5, such as performance on the Orthopedic In‐Training Examination or answers to common questions regarding Tommy John surgery or osteoarthritis, found that ChatGPT 4.0 consistently outperformed the older software in all facets [6, 17, 22, 33]. Furthermore, a study assessing ChatGPT 4.0 responses to questions regarding hip arthroscopy found most of their responses to be ‘excellent,’ similar to the results of this study [28]. This suggests the responses used in this study are likely comparable in accuracy and quality seen in prior assessments of ChatGPT 4.0.

Advantages of ChatGPT for patients are the programme's simplicity and rapid speed at which it produces answers. One question entered can return a lengthy response generated in less than one minute. Another thing to note is ChatGPT's continuous improvement. As previously stated, ChatGPT 4.0 consistently outperforms ChatGPT 3.5 in studies performed in the field of orthopaedics; however, this holds true in a variety of areas including progressing to the capability of passing the bar exam, USMLE Step exams, SATs, and Specialty Certificate Examination in Dermatology, among others [16, 17, 30, 34]. This could suggest that while ChatGPT 4.0 currently generates a range of ‘excellent’ to ‘satisfactory’ responses written at elevated reading levels, future versions may be capable of producing only excellent and easily understood answers.

This study was limited by the subjective grading of responses by the reviewers. However, reviewers were blinded from each other's response grades. Furthermore, both reviewers in the study are fellowship‐trained orthopaedic surgeons with multiple years of experience performing PAO, adding credibility to their evaluations. Second, the questions included in this may not comprise all of the common questions relating to PAO. Still, the two reviewers chose the questions based on their clinical experience. Finally, while we did not use a validated scale to grade the responses, the grading system utilised has been previously applied to assess responses from ChatGPT regarding various orthopaedic topics.

## CONCLUSION

ChatGPT 4.0 offered excellent or satisfactory answers to the most common questions surrounding PAO. Although some patients may find the information hard to fully comprehend due to most responses being written at an advanced reading level, asking ChatGPT 4.0 to simplify or respond at a specific reading level may increase the readability. The 4.0 model has shown the potential to be a valuable adjunct for patient education, though the readability may need to be improved via simplified responses to be useful for the average patient.

## AUTHOR CONTRIBUTIONS

John M. Gaddis, Elias Arellano, Yossef Alsabawi, Pablo Castaneda, and Joel E. Wells contributed to the conception of the study. John M. Gaddis, Blake C. Martin, Elias Arellano, Yossef Alsabawi, Pablo Castaneda, and Joel E. Wells contributed to the study design. John M. Gaddis, Yossef Alsabawi, and Martin Salgado‐Flores contributed to the acquisition of study data. Elias Arellano, Martin Salgado‐Flores, and Charles South contributed to the interpretation of data. John M. Gaddis, Elias Arellano, Blake C. Martin, Yossef Alsabawi, Martin Salgado‐Flores, Pablo Castaneda, and Joel E. Wells drafted/revised the manuscript.

## CONFLICT OF INTEREST STATEMENT

The authors declare no conflict of interest.

## ETHICS STATEMENT

None declared.

## Supporting information

Supplementary Table 1.

## Data Availability

All data was obtained using ChatGPT 4.0.
