# Supplementary material for: Assessing the accuracy and readability of ChatGPT 4.0's original and simplified responses to common patient questions regarding periacetabular osteotomy
Source: J Exp Orthop. 2025 Oct 9;12(4):e70457. doi: 10.1002/jeo2.70457 (PMC12509468; doi:10.1002/jeo2.70457)
Supplement: Supplementary file 1 — Supplementary Table 1. [file JEO2-12-e70457-s001.docx]

**Supplementary Table 1**. Ratings by Question and Reviewer.

|  | | | |  |  |  |
| --- | --- | --- | --- | --- | --- | --- |
|  | Original | | Simplified | | Sixth Grade | |
| Question | Reviewer 1 | Reviewer 2 | Reviewer 1 | Reviewer 2 | Reviewer 1 | Reviewer 2 |
| What is hip dysplasia? | 1 | 1 | 1 | 1 | 1 | 1 |
| What is a PAO, periacetabular osteotomy? | 1 | 2 | 1 | 2 | 2 | 2 |
| Why does hip dysplasia hurt? | 1 | 1 | 1 | 1 | 1 | 1 |
| Do I have to have surgery if I have hip dysplasia? | 2 | 2 | 1 | 1 | 1 | 1 |
| What is the recovery timeline of a periacetabular osteotomy? | 1 | 1 | 1 | 1 | 1 | 1 |
| How soon will I be able to return to physical activity? | 1 | 1 | 1 | 1 | 1 | 1 |
| What are the most common complications related to periacetabular osteotomy? | 1 | 2 | 1 | 1 | 1 | 1 |
| How often do complications occur after periacetabular osteotomy? | 1 | 2 | 1 | 2 | 1 | 1 |
| What is the likelihood of infection after periacetabular osteotomy? | 1 | 1 | 1 | 1 | 1 | 1 |
| What is the likelihood of a fracture after periacetabular osteotomy? | 1 | 3 | 1 | 3 | 1 | 3 |
| How frequently is chronic pain experienced after periacetabular osteotomy? | 2 | 2 | 1 | 1 | 2 | 2 |
| Do the screws get taken out after periacetabular osteotomy? | 1 | 1 | 2 | 1 | 1 | 1 |
| Do the screws placed in periacetabular osteotomy cause discomfort? | 1 | 1 | 1 | 1 | 1 | 1 |
| Can all physical therapists treat periacetabular osteotomy patients? | 1 | 2 | 1 | 2 | 1 | 2 |
| What rehabilitation supplies are needed after a periacetabular osteotomy? | 1 | 1 | 1 | 1 | 1 | 1 |
| How long will I need physical therapy following periacetabular osteotomy? | 1 | 1 | 1 | 1 | 2 | 1 |
| What exercises and stretches help recovery the most following periacetabular osteotomy? | 2 | 2 | 1 | 1 | 1 | 2 |
| What supplements help in recovery after periacetabular osteotomy? | 1 | 1 | 1 | 1 | 1 | 1 |
| What is the likelihood of needing a total hip arthroplasty after periacetabular osteotomy? | 1 | 2 | 1 | 1 | 1 | 1 |
| Will I be able to give birth vaginally after a periacetabular osteotomy? | 1 | 1 | 1 | 1 | 1 | 1 |
| How much does a surgeon’s experience change the outcome of a periacetabular osteotomy? | 1 | 1 | 1 | 1 | 2 | 1 |
| What are the most common patient-perceived complications of a periacetabular osteotomy? | 2 | 2 | 1 | 2 | 1 | 1 |
| What are the reasons for concomitant hip arthroscopy with periacetabular osteotomy? | 2 | 2 | 2 | 2 | 1 | 1 |
| What are the indications for recommending a periacetabular osteotomy? | 1 | 2 | 1 | 1 | 1 | 1 |
| How do I prepare for a periacetabular osteotomy? | 1 | 1 | 1 | 1 | 2 | 1 |
| What is the outcome success rate for periacetabular osteotomy? | 2 | 2 | 1 | 1 | 2 | 2 |
| Is there anything that can improve my pain while waiting for a periacetabular osteotomy? | 1 | 1 | 2 | 1 | 1 | 1 |
| Is undergoing a periacetabular osteotomy worth it? | 1 | 1 | 2 | 1 | 1 | 1 |
| Will I still have pain in my hip after surgery? | 1 | 1 | 2 | 1 | 1 | 1 |
| Will the pain be worse after surgery than before surgery? | 1 | 1 | 1 | 1 | 1 | 1 |

The filled boxes represent discrepancy between reviewer ratings.
